# Factors influencing eating behavior and dietary intake among resident students in a public university in Bangladesh: A qualitative study

**DOI:** 10.1371/journal.pone.0198801

**Published:** 2018-06-19

**Authors:** Ashraful Kabir, Shahgahan Miah, Asraful Islam

**Affiliations:** 1 Dushtha Shasthya Kendra, Dhaka, Bangladesh; 2 Department of Anthropology, Shahjalal University of Science and Technology, Sylhet, Bangladesh; Institut de recherche pour le developpement, FRANCE

## Abstract

**Background:**

Over the past decades, Bangladesh has made substantial progress in improving higher education, and in part, this was achieved by promoting residence based higher education in public universities. University residency is considered a crucial period for students to develop healthy eating habits and adopt nutritious intake, which comprise a strong foundation for good health throughout life. Although, there is extensive literature on eating behaviors and dietary intake internationally, there appears to be relatively scarce research and analysis concerning Bangladesh. This study aims to address this, by investigating the factors that influence eating behavior and dietary intake.

**Methods:**

Adopting a qualitative approach, we conducted 25 in-depth interviews and 13 focus group discussions with students of various disciplines and semesters. We used thematic analysis to analyze the textual data, and methodological triangulation to validate the information provided.

**Results:**

Student eating behavior and dietary intake are influenced by a variety of factors. Individual factors (cooking skills, food taste, food taboos, and knowledge and perceptions), societal factors (influence of peers and social norms), factors related to university (campus culture and frequency of examination), and environmental factors (availability of cooking resources and facilities and food prices) emerged as the key aspects that determine students’ eating behavior and dietary intake.

**Conclusion:**

This is the first study that explored factors influencing nutritional behavior and dietary intake among resident graduates in a Bangladeshi university. The results suggest that resident students have a poor dietary intake that might have a harmful impact on their health, well-being, and academic performance. Therefore, multilevel nutritional interventions may be beneficial to promote healthy eating behavior and dietary intake among university students.

## Introduction

Eating behavior is defined as “normal behavior related to eating habits, selecting foods that you eat; culinary preparations and quantities of ingestion” [1]. Eating behavior is an important aspect of life as it can affect long term health outcomes because unhealthy eating habits such as consuming nutrient deficient food, skipping meals, and a lack of timely diet are understood to cause various health problems and nutritional deficiencies [2;3]. In contrast, a balanced diet and the consumption of quality food can contribute to sustaining the physical well-being and mental stability of individuals [4;5]. Likewise, a healthy diet is understood to play a significant role in the lives of university students—that is, a considerably large population group which could be targeted to prevent numerous health problems [6]. Indeed, international research has demonstrated that student life in the university setting is characterized by many changes in eating behaviors and dietary patterns [7;8].

Over the past decades, Bangladesh has made substantial progress in expanding higher education facilities (with an inclination towards public universities). In order to enhance higher education in multifaceted disciplines and maintain international standards, the government, in principle, has favored initiatives that build new public universities in the headquarters of each of the older districts (which are large in population density and in area). The University Grant Commission of Bangladesh reported that 129 universities are operational in the country, of which 92 are private and 37 are public universities [9]. Usually, public universities promote on-campus residential facilities and students are typically accommodated in university dormitories (commonly known as halls).

Such residential facilities provide a wonderful and hands-on experience to freshmen in many aspects of their lives as they spend a substantial length of time in universities, especially students from lower socio-economic backgrounds and rural settings. In many cases, the university living environment placed freshmen in unusual circumstances involving detachment from family members, new friends and roommates, student politics, and campus norms and culture, as they enter universities with excitement and perhaps, in some cases, struggle to adapt to this new environment [10;11]. Eating patterns and dietary habits are likely to be an additional challenge for students as they negotiate the many difficulties of the new environment [12]. Previous studies indicate that eating behaviors and food habits are determined by a variety of individual, social, and environmental factors of the given context [13–15]. Globally, the literature suggests that during the transitional period from secondary level to graduate level, students often engaged in unhealthy dietary habits and had poor nutritional intake [15–17]. However, adequate nutrition is fundamental at this age in order to maintain good physical and mental health, ensure healthy cognitive and intellectual development, and achieve optimum academic performance [18;19].

There appears to be a paucity of research on university student eating behaviors and dietary patterns in Bangladesh as compared to the substantial literature which has been produced internationally. Considering the scarce evidence in Bangladesh, this study is aims to investigate the factors that influence eating habits and dietary intake of university students. This study will serve to tend to this knowledge gap and thus contribute helpful evidence for program managers and policy planners to improve these factors. In this study, we adhered to the trustworthiness by applying four principles—credibility, transferability, conformability, and dependability. We used inter-coder or synchronic reliability that refers to the amount of agreement between impendent coders of the data. We measured the agreement during the analysis when the researchers’ coded same interviews independently. We furthermore performed triangulation between methods, and participants.

## Methods and materials

### Study time and settings

This study was conducted between January and December 2016 in Shahjalal University of Science and Technology—one of the reputed public universities located in the periphery of Sylhet metropolitan city, Bangladesh. It is nearly 240 km from Dhaka, the capital city of Bangladesh, and is connected by highway, railway, and airway. The university was established in 1987, pioneering science and technology education as a specialized state-supported institution in Bangladesh. The university possesses an area of 320 acres. Currently, it hosts approximately 9,000 students in different courses, and provides residential facilities for the students in five dormitories on the campus; and it has three additional female hostels outside the campus run by the university authorities privately (rental houses where students have to pay higher rent than those on campus). Three of these on campus are allocated for male and the rest for female students. Each of the dormitories has a cafeteria. Additionally, there are many canteens, cafeterias, grocery stores, and restaurants on the campus. These privately-owned shops and restaurants became the key places where food and beverages are prepared and served to the students.

### Study population and sample

Participants who were recruited consisted of students enrolled in their first year and fourth year of study under various departments. Inclusion criteria required that participants were aged between 17 and 25 years old and participated voluntarily. In this process, we invited students who showed a proactive interest in sharing their experiences, practices, opinions, and time. We interviewed resident students who had experience in cooking and eating in the dormitories. They were from various parts of the country and from different dormitories. Considering the study objectives, this study adopted non-random sampling strategy in recruiting the participants. The participants were purposefully recruited for the interviews concerning their eating behavior, experiences, practices, and perceptions to investigate which factors influenced dietary intake and eating behavior on their campus life and how these factors did so. We determined the sample size following the principle of data saturation—reaching a point where no new data and/or theme and/or dimension emerged in the interviews [20]. In order to validate the data collected a number of processes were utilized—methodological triangulation, and the purposeful and gradual sampling and selection of the participants. We conducted a total of 25 in-depth interviews (IDIs) and 4 focus group discussions (FGDs). We conducted IDIs to understand individual cooking and eating experiences, practices, and behavior; while FGDs provided data to understand and clarify students’ opinions, beliefs, attitudes, behaviors, and perceptions on the topics. In each FGD, we included 6–8 participants, which is considered to be an ideal sample size for such qualitative research [21]. All interviews were conducted in private locations—i.e.—in an empty classroom and/or corner of the playground.

### Data collection procedure

A research team of four members who graduated in anthropology and public health conducted the IDI and FGD. Two researchers (first and third authors) conducted IDIs, while the second author facilitated FGDs. The research team was trained in qualitative research methods and had extensive field experience. All IDI and FGD were conducted in the Bangla language—the mother tongue of the participants and interviewers. We developed interview guidelines to explore a set of topics relating to various issues that influence eating behavior and dietary intake. In order to gather information in a semi-structured and systematic manner, we developed an interview schedule. This document was used to guide conversations around key dimensions relevant to our research questions and objectives, including socio-economic and demographic issues that impact eating behavior and dietary intake among the students. Interviews were semi-structured in order to create a friendly rapport with respondents and leave sufficient space for other themes to emerge. Open-ended questions were used to explore the individual, societal, environmental, and university related factors that affect their cooking and eating habits, eating hours, and the dynamics of different factors. It took approximately 45–60 minutes to conduct each IDI and 90–120 minutes to conduct each FGD respectively. The interviewers established a good rapport with the participants before conducting the discussions. For some cases, several follow-up visits were made to gather missing data and to further probe some issues. Detailed field notes were prepared during the interviews. Verbatim transcriptions were performed for all IDI and FGD and all the interviews were electronically recorded. Following the transcription, the texts were translated into English, and in order to ensure the quality of the translation—that the translation was consistent with the original—the researchers double checked the translations to avoid errors.

### Data analysis

We used thematic analysis to analyze the textual data [22] and the analysis was conducted manually (i.e.—no textual software was used (e.g. Atlas-ti or Nvivo). Open codes were generated, and then we coded the text of all the transcripts through repeated readings. After all the initial coding of the interviews were performed, we looked for clusters of several codes—termed “themes” or “concepts.” In order to increase the validity of the coding system, triangulation of researchers and methods were performed by the authors independently coding a few transcripts, and then establishing a consensus after discussion.

### Ethical issues

This study was granted ethical approval by the institutional review board of the “Shahjalal University of Science and Technology.” We developed an informed consent form to explain the study objectives and its importance, confidentiality, anonymity, potential loss and benefit, and participant rights. The written consent form was read out and the participants were encouraged to ask further queries concerning the interview process. We obtained written consent before conducting each interview. We collected personal data from the participants including age, sex, education, department, hall of residence, and religion. However, we maintained confidentiality of personal identification of all participants—access to and use of the records and data was restricted to only the research team—and ensured that the data was stored in a secured location. A participant identification (ID) number was generated for each participant as a precautionary measure and these ID numbers were removed prior to reporting the findings.

## Results

In terms of the sociodemographic characteristics of the sample, the data indicated that the participants were between the ages of 17 and 25 years old. Table 1 shows that the mean age of the IDI participants was 21.4 (SD±2.2) years; while, it was 22.6 (SD±2.6) years for FGD participants. Among IDI participants, the majority of students were in their second year (7 out of 25) and third year of study (8 out of 25); whereas, most students in FGD were in the fourth year (10 out of 26). The highest numbers of participants in the IDI (7 of 25) and FGD (6 of 8) are students of both the social sciences and physical sciences. In both methods, a higher number of the participants were male (16 of 25 in IDI and 16 of 26 in FGD) and the majority of participants were from rural settings and from families following Islam.

**Table 1:** Socio-demographic characteristics of the participants (IDI: n = 25, FGD: n = 4).

Characteristics	Methods	
	IDI (n = 25)	FGD (number of participants; n = 26)
Age in years (mean ±SD)	21.4±2.2	22.6±2.6
**Academic year of study**		
First year (*n*)	4	4
Second year (*n*)	7	8
Third year (*n*)	8	4
Fourth year (*n*)	6	10
**Major of study**		
Social sciences (*n*)	7	6
Business studies (*n*)	3	2
Applied sciences & technology (*n*)	5	4
Life sciences (*n*)	3	6
Physical sciences (*n*)	7	8
**Gender**		
Male	16	16
Female	9	10
**Family residence background**		
Rural area (*n*)	18	18
Urban area (*n*)	7	8
**Religious identities**		
Muslim (*n*)	17	16
Hindu (*n*)	6	8
Others (*n*)	2	2

**Fig 1:**
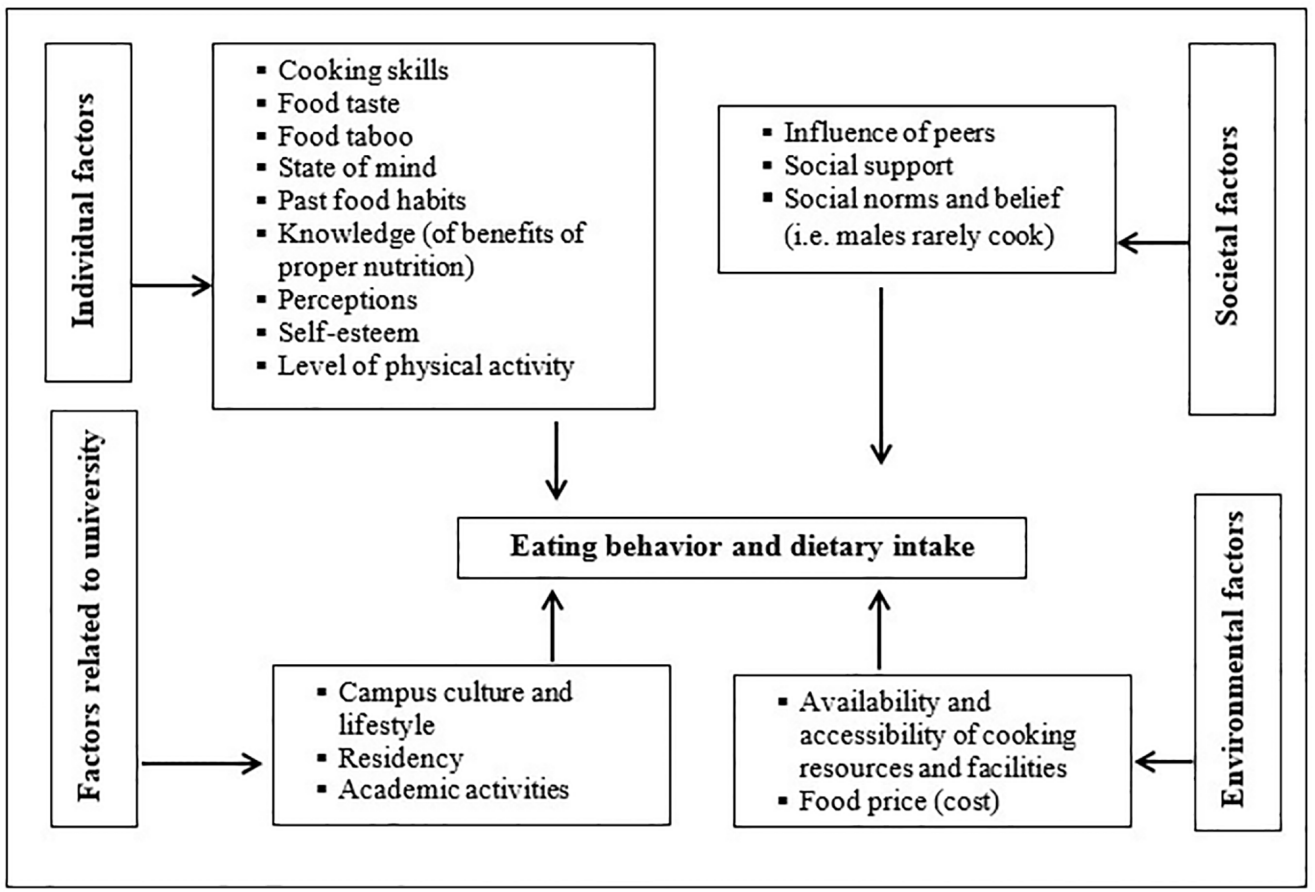
Emerging codes and factors.

In terms of the thematic analysis, our data revealed that there are four major factors determining the eating behavior and dietary intake of resident students in the university, including individual factors, societal factors, environmental factors, and factors related to the university (Fig 1). Each of the factors are further is divided into sub-factors. Our data revealed that the vast majority of students frequently consumed food low in nutritional value from on-campus food vendors based on food prices, availability, food quality, and the physical environment (Table 2).

**Table 2:** Patterns of common food and its consumption by students.

Type of meals	Sources	Common items	Availability (time)	Reason for food choices
Breakfast	Dining halls, canteens	*Khichuri* (made from rice and lentil), egg (omelet, curry), *Alu Bhorta* (mashed potato)	8.00 am–11.00 am	Comparatively low cost
	On-campus food courts, mobile vendors,	*Khichuri* (made from rice and lentil), banana, *Pakora*, *Singara*, *Somosa*, *Alor chop* (snacks made with potatoes, vegetables, boiled eggs, traditional spices, and tamarind sauce), Noodles, Vegetable roll, Burger, Sandwich, Fruit juice (based on seasonal fruits), Coke, mineral water, tea	8.00 am–10.30 pm	Available in early morning, long duration for food availability
	Campus *tong* (small shop)	*Khichuri* (made from rice and lentil), egg (omelet, curry), mixed vegetables, *Poratta* (flatbread made from flour), fish, chicken, tea	8.00 am–7.00 pm	Comparatively better quality, availability of tea, reasonable price, gossiping and smoking space
Lunch/dinner	Dining halls, Canteens	Rice, lentil, vegetable, fish/chicken, beef (very rare)	12.30 am–3.00 pm	Good seating arrangement, different options, low price
Daylong	On-campus food courts, mobile vendors	*Chotpoti*, *fuska*, (made from potato, spice, and tamarind) *jahlmori (puffed rice with spice)*, *papor*, pickle, nuts, *labor shorbot* (a drink made with sugar cane, lemon leaf, and ice), ice-cream, *pitha* (traditional pancake), boiled egg	10.00 am–8.00 pm	Diverse items, low price, daylong availability, time saving

### Individual factors

#### Cooking skills

Individuals’ cooking skills emerged as a factor influencing dietary intake on campus life. The students who had cooking skills tended to have made cooking arrangements in their rooms. On the other hand, those who did not how to cook avoided cooking in their personal rooms. The participants reported that despite the poor quality of diet served in the dining hall the students who lacked cooking skills had to adapt to it. However, some of the newly admitted students were reported to develop their cooking skills gradually and tended to make cooking arrangement on their own. Overall, female students had better cooking skills than their male counterparts. Possibly the female students learnt these skills at their homes. One participant stated this in the IDI:

“In Bengali culture, female individuals are usually familiar with cooking activities. Hence, they gain good cooking skills within their family environment. …female students are commonly seen to cook in their personal living arrangements. …but you see the percentage of students who cook is much less among the male students than the female.”

(First year female student)

The opposite perception was reported about the male students’ cooking skills. One participant of FGD stated this:

“Male individuals rarely participate in cooking in their adulthood. They usually learn cooking in halls by doing it.”

(Second year male student)

#### Food taste

Food taste emerged as a common factor for choices concerning food intake among the participants. The participants admitted that color, smell, and texture of food were usually considered in food choice. Although the participants mentioned these aspects commonly, they were found to be variably inclined to choose based on their familiarity and experiences that are often linked to regional backgrounds. For example, some of them favored spicy items; similarly, some of them preferred sweetness or bitterness. However, almost all respondents stated that food items served at the canteen of residence halls are monotonous in taste as they use the same ingredients and raw elements throughout the year. Therefore, the graduates often are seen to make some personal arrangement in preparing dishes in their rooms. Some of the participants even sought meals in restaurants outside the campus for a change in taste. The situation was explained in the following quote from an FGD:

“Yes, students consider taste in choosing food items. Taste includes color, smell, and texture of the dishes that you are eating. To avoid monotonous diet, students often enjoy dishes from outside the campus.”

(Third year male student)

#### Food taboo

Eating behavior and eating patterns based on food taboos were reported. Many students reported deliberately avoiding some food items which are prohibited by their own religion. For example, students following Hindu religion never consumed beef, as their religion prohibited it. One participant explained this:

“Students strictly maintain food taboos. They never consume items that are not permitted by their religion.”

(Fourth year male student)

#### State of mind

State of mind was commonly reported to be a notable factor affecting the eating habits and choice of dietary intake. Stressful conditions such as exam pressure influenced students to compromise on food quality. Students showed no or little interest in cooking ahead of the exam. Some of the students even skipped breakfast or failed to take delayed meals. On the other hand, they were found to take meals in restaurants, while they prepared for exams. The students tended to celebrate the end of their exam by exploring different dishes and tastes in and around the campus. The situation was explained in an IDI:

“When you finish an exam, it’s a great thing in your life. You can celebrate that event by exploring some taste.”

(Fourth year male student)

Similarly, one participant of an FGD said this:

“Before the exam, you cannot think about anything else. Food quality is not a big issue; rather, how to pass the exam is.”

(First year female student)

#### Past food habit

Past dietary habits of students influenced their eating behavior and food choices on the campus environment. Students seemed to be inclined to consider taste, color, and ingredients of food with which they have been habituated, during their family life. Ultimately, family feeding practices influenced students who became inclined or disinclined to choose particular types of food. In general, the persons who came from rural settings seemed to include diets with carbohydrates (plain rice) and spicy items; whereas, students from urban backgrounds were more inclined to include non-carbohydrates (processed food) and non-spicy foods in their diet. One participant elaborated on the issue in an IDI:

“I am from a rural setting. I am used to eating rice since childhood. If I eat all the food available in the world except rice, I feel I did not eat for days. I cannot survive a single day by avoiding rice.”

(Second year male student)

#### Knowledge and perception about nutritional benefit

The level of knowledge and perception about the health benefits of nutrition influenced the eating behavior and dietary intake. Most of the students had limited knowledge about the health benefits of nutritious food items. A large number of participants reported that consuming food is the key issue, regardless of the type of food. The majority of the participants did not consider the nutritional value while consuming a particular food item. They even admitted that they did not have good knowledge about the nutritional value of the food they commonly eat as part of their daily meals. One of the participants reported in an IDI:

“In my meal I just consider those items with which I have been habituated to for long. I do not know what nutritional component is contained in it and which one is beneficial for my body…I do not know which food contains which nutritional component, which foods provide proper nutrients and are balanced in proteins, carbs, vitamins, fats, and minerals.”

(Third year male student)

Moreover, most of the health benefits of proper nutrition were perceived to have little/no importance among the participants in all methods. The participants believed that considering nutritious and balanced food intake might not be a significant issue at such a young age. The health conditions of the young graduates were believed to be good as they maintained a routine which included their studies. Some of the participants assumed that they were in good health as they were not yet diagnosed with any illness, and thus appeared to infer that their nutritional needs were met, regardless of what they ate. Such notions were explained by the following quote in an FGD:

“I am a healthy man since I do not have any disease. Why should I worry about the nutrition components of food now? Moreover, I can maintain my daily activities without feeling weak.”

(First year male student)

Similarly, another participant said this in the IDI:

“Maintaining nutritional needs of the body is unlikely to be a crucial issue in young age. You can maintain such a health condition that is needed to live a sound life because of your age. Dietary intake is likely to be to a vital concern for the elderly people.”

(Fourth year female student)

#### Level of physical activity

Level of physical activity appeared as a factor in determining dietary intake. Students engaging in the habit of regular physical exercise seemed to prefer larger quantities of food intake than those who did not have any physical activity. Physical activities such as outdoor sports, body building, and regular walking influenced dietary choice—mostly involving a larger quantity of carbohydrates and protein intake. The participants believed that physical activity leads to a loss of energy and higher amount of carbohydrates are important for balancing this loss of energy. One participant stated this in the FGD:

“If you perform physical exercises such as jogging, you lose much energy. …you must find a balance between the damage and reparation of energy. Larger amounts of carbohydrates such as rice, along with proteins such as beef or egg can be a good source of this reparation as it is available, cheap, and easy to cook.”

(Fourth year male student)

However, few participants thought that carbohydrate and protein intake alone may not be enough to reenergize the body, and that vitamins, minerals, and vegetables are also the vital. Nevertheless, the students could not manage to consume it is as much as they need, due to the high costs and difficulty of preparing them. For example, although vegetables and fish are cheap and available in markets, it is difficult to process and cook them. One participant explained this situation in an IDI:

“If I cook meals I prefer beef or egg as a source of protein. I know that fish and vegetables are good sources of proteins and vitamins as well, but I avoid it because it’s very difficult to prepare. …again, if I have food from the hall canteen, I cannot taste a variety of vegetables as they offer a single vegetable in a meal.”

(First year male student)

### Societal factors

#### Influence of peers

Hall inhabitants, roommates, course mates, close friends, and classmates were reported to be consistently influential in the participants choice of dietary intake. Participants in all methods informed that when a few students are arranging for meals together, they preferred to cook two or more dishes with curry and were also found to be cooking regularly as it was easy. They tended to be accustomed to preparing a variety of dishes including meat/fish, vegetables, rice, salads, and beverages (occasionally). One participant illustrated this factor in an IDI:

“A few students prepare meals together, as it costs less and requires little time and labor. Subsequently, they can prepare a variety of meals. …thus they avoid monotonous dishes.”

(Third year female student)

Similarly, when a few friends/course mates had food from on-campus canteens, they were inclined to have several dishes as they shared the costs between them. On the contrary, while some students prepared meals separately they tended to only have a limited variety of dishes to avoid the intense labor, time, and cost. In such cases, these students preferred to consistently have meals with rice, mashed potatoes, and eggs. One participant in the FGD said this:

“If you are alone, everything is done by you, including shopping, preparing, cooking… you are subsequently limited to a single dish… most often you prefer to cook dishes which are easy to prepare with low costs, such as mashed potatoes and rice”

(Third year female student)

#### Social network

Participants revealed that social interactions and personal interactions were a factor influencing their food choices and dietary intake. Students who had a wider friendship network on the campus and/or hall of residence reported having celebrations involving food in famous restaurants in Sylhet city. Students often tried to test dishes in restaurants in the nearby city in a group as they could share the cost between the group members and have a variety of delicious dishes. One participant mentioned this in the FGD:

“I have good friendships with a number of students in different disciplines. We test different dishes in famous restaurants such as Panshi or Kutum bari very often. We can afford it because we share the dish and cost.”

(Second year male student)

#### Social norms and beliefs

Female students more frequently reported having the habit of cooking than the male students. In *Bengali* culture, male individuals rarely participate in cooking activities. However, females were expected to learn cooking throughout their childhood and adolescent years and are therefore likely to have experience in cooking and tended to cook for themselves in their residence hall. In many cases, a group of female students collectively cooked a single meal for themselves. Female students were found to like fresh food and a variety of dishes compared to the male students. One participant said in an IDI:

“Naturally, female students are familiar with cooking. They learned it during their childhood. Therefore, they might not get bored with cooking. …of course they will get fresh and a variety of dishes as they know how to cook?”

(First year female student)

### Environmental factors

#### Availability and affordability of fresh raw food

The availability of fresh raw food items with affordable prices appeared to be a considerable factor determining the diet. Participants reported that food, especially seasonal vegetables (winter), with affordable prices favor cooking in the hall of residence. This trend was mostly found among female students. During winter, a lot of vegetables become available on the campus premises at low prices, influencing their cooking arrangements; therefore, the consumption of vegetables increased in winter. Similarly, in monsoon, the students found cooking somewhat difficult as the availability and affordability of vegetables reduced. Besides, in the monsoon, acquiring fresh and raw food ingredients seemed to be difficult on campus. Considering the unavailability and higher price of raw food items, students tended to avoid cooking and were inclined to eat at dining halls and/or canteens and mobile/temporary food shops on the campus. One participant told in an IDI:

“Due to the availability and low price, students become interested in cooking in the winter. However, they avoid it in the rainy season, as they lack adequate vegetables in this time. But, in winter, they consume adequate vegetables.”

(First year male student)

Vegetables become available and popular in the hall canteen and temporary food shops in the winter. The students tended to consume more vegetables in that period than in other seasons.

#### Food cost

Food cost was commonly mentioned as a significant issue in choosing and/or arbitrating dietary intake among the students. The data revealed that many students skipped breakfast after considering the cost. A substantial number of students had the habit of having lunch instead of breakfast intentionally so they can save 15–30 taka. (USD 0.2–0.4). As most students in the public university hail from the lower middle socio-economic class; they received a meager amount of money from their homes to survive. Consequently, they tended to adopt a coping up strategy by cutting expenses incurred on breakfast. One participant stated in the FGD:

“Many of us are from poor and middle class families. Thus we receive no/little money from our families. As a coping mechanism, we sometimes skip breakfast intentionally.”

(Third year female student)

On the other hand, some participants revealed that many students habitually slept late at the night due to studying and/or other activities (gossiping/watching movies/watching sports on a PC or TV). Consequently, they had some light snacks (including biscuits, dry cake, tea, and coffee) before sleeping. They usually woke up late (after 10 am) and did not want to have breakfast. Often, they had to hurry to attend class. In many cases, they preferred to have lunch directly instead of breakfast. One participant informed in the IDI:

“Those who slept late usually woke up late. In the midnight, they usually had some snacks which energized them till lunch in the following day. Thus, they could avoid breakfast.”

(Second year male student)

Nevertheless, a few participants revealed that sleeping late and avoiding breakfast had become the hall culture. Many students who had the habit of sleeping late did not have snacks before their sleep. Therefore, they were more likely to have a nutritional deficiency. They probably likely had little or no knowledge of the importance of having breakfast.

### Factors related to university

#### Campus culture and lifestyle

The participants expressed that their peers, roommates, and friends had a big influence in the choice and arrangement of dietary intakes. Students, who regularly engage in sports and physical activities such as athletics, body building (i.e.—visit gymnasium), cycling, and playing football and cricket are inclined to consume food including vegetables and proteins. They believed that rice is the key source of carbohydrates which causes people to become overweight and were obsessed with diet; and therefore, they tried to keep it out of their diet. Instead, they tried to consume a diet containing proteins and iron. Eggs and chicken were reported to be the most common food items that were considered as a good source of protein that had a low cost. One student mentioned in the IDI:

“I go for sports in the afternoon on a regular basis and I know that my body loses a lot of energy. I try to energize by having more nutritious food. …rice contains very low nutrients that may result in gaining weight. But, an egg or a banana may have benefits with low costs.”

(First year male student)

However, the participants commonly reported that very few students engaged in regular physical exercises or sports. Those who do not get involved in sports seem negligent of the importance of consuming a nutritious diet and/or regular and timely meals.

#### Residency

Students who are from a locality (near the university) frequently visited their homes in the weekend and had the opportunity to have a nutritious dietary intake. Some participants reported that students living in the residence halls where there were good cooking facilities were more likely to engage in cooking. In most cases, residence halls for female students had good cooking facilities such as gas burners (two burners on the ground floor) and electronic heaters, which motivated the students to cook. However, the senior students (fourth year/master students) were reported to enjoy these facilities more than the junior and/or new students. Besides, students from those residence halls where the vendors sold vegetables were reported to engage in cooking and consuming more fruits. One participant from IDI specified this:

“Usually vendors sell vegetables and fruits such as banana, water melon, pineapple, guava, and seasonal fruits near some halls. Those living in these halls have more opportunities to have lost of vegetables and fruits. …they are even motivated to try diverse dishes.”

(Fourth year male student)

#### Academic activities

The participants reported that students tended to avoid cooking during and before exams. They spent a greater time preparing for exams. They consistently invested efforts and time till midnight to late night. During their study, students consumed more liquid and processed food such as tea, coffee, and snacks. Those who used to cook tended to avoid it due to the examination. Some of the participants reported that if they felt pressured due to the specific examination (e.g. semester final, tutorial, assignment), they avoided meals. Moreover, they were likely to irregularly consume meals. Due to exam pressures, students tended to have more processed foods as it was easily available and could be preserved in their rooms. One participant explained this situation in an FGD:

“Students invest a lot of effort in preparing for exams. Intensive study and exam pressure places them in a situation where they spend sleepless nights. During that time, they need extra calories that can energize them. Processed food is the best source that can provide some calories at midnight. …if you have some biscuits or bread or a banana, if can satisfy your need during the study. … and many of us do so.”

(Fourth year male student)

On the other hand, if students feel relieved of the exam pressure, they celebrated it by going to restaurants and/or arranging a little feast.

## Discussion

To the best of our knowledge, this is the first study that explored a range of factors influencing nutritional behavior and dietary intake among the resident students in a Bangladeshi university. Accordingly, the existing literature presents very little information about how and whether food habits and food intake are influenced by various factors among students in public universities in Bangladesh. Our analysis revealed that a broad spectrum of factors influenced the eating habits of resident graduates and possibly led to poor nutritional intake among them. Data from different methods commonly supported the views that these factors are interconnected and influence each other (Fig 1). Individual cooking skills, food tastes, costs, habituation to certain dietary intakes, perception and knowledge of nutrition benefits, peer influence, campus life style, exam pressure, and availability of cooking equipment were the key reported factors that determined eating behavior and dietary intake.

Our findings showed that a large number of male students were more likely to have food from food courts, shops, and mobile vendors on the campus, especially during the daytime. This trend might be explained by the fact that a lack of cooking skills influenced male students to be more prone to consume commercially prepared food from these sources within the campus premises. Usually, food vendors offer monotonous food items which are poor in quality [23]. Along with limited cooking skills, limited access to resources for cooking and the poor standard of living conditions notably limit the likelihood of the meal frequency which is suggested by the existing study [24].

Additionally, in concordance with the global literature, our study showed that a vast majority of students are likely to skip meals; and especially, many of them deliberately avoid breakfast. Internationally published studies conducted in Saudi Arabia, Malaysia, Turkey, and Nigeria offer evidence that university students avoided meals due to various factors, including individual, societal, and environmental reasons [6;25;26]. However, this trend of skipping breakfast might be explained by economic aspects of the students in our study. As the majority of the resident students belong to lower middle-class families, and thus have a limited financial capacity to afford food. A study conducted in other settings presents similar views that socioeconomic capacity is associated with food habits; for example, adolescents in schools in Nordic countries are more likely to consume fruits and vegetables daily [27].

Preference for tastes and family eating habits were notably mentioned by many participants as influential in determining dietary intake. This is in line with other studies that present similar views; for example, Drewnowski et al. [28] suggested along with social and cultural factors, taste preference plays a central role in food selection. Further, Drewnowski believed that food taste, food preference, and food selection and consumption are interlinked and thus influenced by each other [29]. Moreover, family eating patterns were observed to be influential in food intake selection as observed by Schnettler in a Chilean study [30].

The level of knowledge regarding the nutritional value of food items played a role in developing eating habits and therefore, in selecting food in their meals. Our analysis showed the students had poor nutritional knowledge which resulted in them consuming more fat-like substance,. This analysis is concordant with other studies; for example, Wyka et al. [31] observed a low level of nutritional knowledge in the agricultural university of Wroclaw where male students had a high intake of fat, meat, and eggs. Another study observed that university students in China possessed poor knowledge regarding healthy diets and developed an increased tendency of eating out [32]. A study conducted among young students in University of Valencia showed that they had a high intake of fat protein content as compared to Spanish recommendations [33]. Physical activities were observed in this study as determining eating habit and dietary properties. The majority of students did not engage in physical activities and lacked motivation to consume a balanced diet; whereas, very few students reported a focus on quality food as they regularly engaged in physical activities including outdoor sports. This analysis is in line with the findings of a cohort study by Downers et al. suggesting physical activities act as strong motivating factors for healthy dietary habits among college students [34].

Our analysis suggested that societal factors influence students’ eating behavior and food choice. Students’ food choice was clearly influenced by their social network (course mates, roommates, and friends) in and around the campus. Previous studies support our analysis; for example, Contento et al. [35] reported that peers influence adolescent food selection as they influence each other’s behaviors, attitudes, practices, and preferences. Other studies reported that socio-environmental aspects such as peers and friends influenced the eating patterns [36]. Existing social norms and beliefs are reported to have remarkable influence on eating behavior and selecting food content. Our data support that female students are more likely to cook in their halls. This trend is possibly supported by the norms that female individuals are expected to have cooking skills and cooking practices as observed by Kabir [37]. As they are less likely to choose processed food, they consume vegetables, fish, and meat items more than male students. Studies conducted in other settings present similar findings that female students are more inclined to have a high intake of vegetable, milk, fish, and fiber than male students [38–40].

The information acquired through our study suggested that many students’ eating habits and dietary intake was notably influenced by academic activities, especially exam pressures. Studies in German universities observed that study pressure reduced the time available for cooking, which lead to unhealthy eating among the students [41]. Another study conducted in the University of Newcastle, Australia, reported that study pressure, together with environmental and university related factors, affected the choice of snacks [42]. More stressful conditions led male students to consume greater amounts of unhealthy processed food in and around the university campus as observed by Papier et al. in an Australian University [43].

Food availability was frequently reported as an factor for choosing food and thus notably impacted the eating behavior as observed by Duarte-Cuervo et al. in Bogota [44]. Our data shows that students were notably influenced by the availability of food and thus heavily inclined to have breakfast and lunch from food vendors on the campus premises as they are available during the day.

## Limitation of the study

One of the limitations of this study is that we adopted purposive sampling technique which is relatively small in size. Another limitation is that this study was conducted in a university which is located outside the city center. In addition, we considered that the lower number of female participants included in our study was a limitation. This occurred because a few female students refused to participate in the interview at the time as they had other engagements they had committed to, such as exams, tutorials, and field work, for example. In addition, some contextual characteristics such as location, size, structure, and local environment might differ from that of other universities. Therefore, the results of this study might not allow for the generalization to other settings. However, the objective of this study was not to generalize results; but rather, to develop a rich understanding of participants’ practice, experiences, and factors determining their eating habits and dietary intake. Moreover, considering the principle of data saturation, this study presents an in-depth understanding of the factors influencing eating behavior and dietary intake of students at a tertiary level educational institution in Bangladesh.

## Conclusion

This study was designed to explore the factors that influence eating behaviors and the consumption of food among the resident students at a Bangladeshi university. It suggests that a broad range of factors (intrapersonal, interpersonal, and environmental) play a vital role in developing eating behaviors and food consumption. Intrapersonal factors such as cooking skills, taste preference, family eating habits, and level of nutrition related knowledge and perceptions impacted the development of eating patterns. Along with these individual aspects, societal and environmental factors including campus culture, lifestyle, peer influence, academic activities, and food prices and availability were found to be influential in determining eating habits. The study also suggests that the knowledge regarding nutrition is poor among the resident graduates. However, campus life should possibly provide the students with crucial opportunities to develop healthy eating habits and adopt a balanced diet which form a strong basis for good health throughout life. Therefore, interventions need to consider individual, societal, environmental, and contextual factors in order to aim at increasing healthy eating behaviors and improving the dietary intake of university students.

## Supporting information

S1 File Guideline for in-depth interview (IDI). (DOCX)

S2 File Guideline for Focused group Discussion (FGD). (DOCX)
