# Supplementary material for: Factors influencing eating behavior and dietary intake among resident students in a public university in Bangladesh: A qualitative study
Source: PLoS One. 2018 Jun 19;13(6):e0198801. doi: 10.1371/journal.pone.0198801 (PMC6007825; doi:10.1371/journal.pone.0198801)
Supplement: S1 File — (DOCX) [file pone.0198801.s001.docx]

Guideline for In-depth Interview (IDI)

1. Socio-demographic information of the participants (name, age, year of study, discipline/faculty/Centre, family structure, income, hall of residence etc.)
2. Would you please say something about eating? (What and how and why do you eat? Where and why do you eat? Who and how serve you? What and how affect your eating etc.?)
3. Please say something about your food selection. What type food does take? How and when you take? Please discuss elaborately (when, how, why, and why not?)
4. In your opinion, what are the important elements/aspects that affect your food choices in and around your university? (Why and why not?)
5. In your opinion, what aspects/issues/elements affect your eating in and around your university? (Why and why not?)
6. How do these elements/aspects affect your health and nutrition? (Why and why not?)
7. In your opinion, how these conditions can be improved? (Why and why not?)
