# Supplementary material for: Factors influencing eating behavior and dietary intake among resident students in a public university in Bangladesh: A qualitative study
Source: PLoS One. 2018 Jun 19;13(6):e0198801. doi: 10.1371/journal.pone.0198801 (PMC6007825; doi:10.1371/journal.pone.0198801)
Supplement: S2 File — (DOCX) [file pone.0198801.s002.docx]

Guideline for Focus Group Discussion (FGD)

1. Socio-demographic information of the participants (name, age, income, gender, religion, length of study, discipline/faculty etc.)
2. How and what do you eat?
3. How is health and nutrition status?
4. In your opinion, what are the important elements/aspects that affect your food choices in and around your university? (Why and why not?)
5. In your opinion, what aspects/issues/elements affect your eating in and around your university? (Please discuss elaborately when, how, why and why not?)
6. How do these elements/aspects affect your health and nutrition? (Why and why not?)
7. What are other important factors that affect your food choices in and around your university (resources, buying capacity/income, firewood, family composition etc.)? Why it is important and why not?
8. In your opinion, how these conditions can be improved? (Why and why not?)
